# Epidemiological investigation of allergic rhinitis in children aged 6–12 years in Bayannur City, China

**DOI:** 10.3389/fped.2024.1422323

**Published:** 2024-09-24

**Authors:** Xiaobo Yan, Limin Li

**Affiliations:** ^1^ Graduate School, Baotou Medical College, Inner Mongolia University of Science and Technology, Baotou, Inner Mongolia Autonomous Region, China; ^2^ Otolaryngology Head and Neck Surgery, Bayannur City Hospital, Bayannur, Inner Mongolia Autonomous Region, China

**Keywords:** allergic rhinitis, children, epidemiology, China, Bayannur City

## Abstract

**Background:**

Allergic rhinitis (AR) is an inflammatory condition of the nasal mucosa triggered by exposure to non-harmful substances. Over the past decade, the prevalence of AR in Chinese children has been steadily increasing. However, detailed epidemiological data on AR in children from Bayannur City are lacking.

**Methods:**

This study randomly selected six primary schools in Bayannur City. Electronic questionnaires were distributed via the web, and parents and children completed the questionnaires by scanning the two-dimensional code within a designated timeframe. Statistical analysis was performed on the collected data.

**Results:**

A total of 4,754 valid responses were obtained. The self-reported prevalence of AR among children in Bayannur city was 39.79%. Multivariate analysis revealed that male gender, belonging to an ethnic minority, a history of food or drug allergies, frequent antibiotic use (≥3 times per year in the past two years, with each course lasting ≥3 days), and residence in urban or pastoral areas was associated with an increased prevalence of AR in children. The proportion of children experiencing moderate to severe AR hat impacted their studies or daily life was 48.78%. Chronic AR was reported in 56.71% of cases. Among AR patients with other allergic conditions, the incidence rates were as follows: bronchial asthma 35.99%, upper airway cough syndrome (UACS) 64.32%, secretory otitis media (SOM) 22.41%, obstructive sleep apnea hypopnea-syndrome (OSAHS) 49.58%, allergic dermatitis (AD) 48.72%, and allergic conjunctivitis (AC) 85.20%. The prevalence of AR was 50.30% in urban areas, 13.733% in rural areas and 20.90% in pastoral areas. Seasonal effects on AR prevalence were notably significant in urban and pastoral regions.

**Conclusions:**

The prevalence of AR among children in Bayannur city was 39.80%. Of those with AR, 48.72% experienced significant impacts on their learning or daily life, while only 14.80% had no other allergic conditions. There were significant variations in the prevalence and onset of AR among children between urban, agricultural and pastoral areas.

## 1 Introduction

Allergic rhinitis (AR) is a non-infectious condition characterized by inflammation of the nasal mucosa in response to exposure to allergens such as dust mites, pollen, dairy products, and eggs (1). The primary symptoms include runny nose, sneezing, nasal congestion, and itchy nose. AR affects approximately 25% of the global population (2), with the majority of individuals exhibiting symptoms before the age of 20, and nearly 50% of patients showing symptoms by age 6 (3). AR not only disrupts daily activities, academic performance, and sleep in children but also increases the risk of physiological disorders such as depression and bipolar disorder in severe cases (4). In children aged 6–12 years, AR can significantly impact sleep quality, facial development, and vocal function (5). Additionally, AR often coexists with bronchial asthma, upper respiratory syndrome, allergic dermatitis, allergic conjunctivitis and other allergic diseases, which collectively impair children's development and physical and mental health.

According to 2019 report, the prevalence of AR in Chinese children was 15.8%, of which the prevalence rates in central China, South China, Northwest China, Taiwan, Southwest China, North China and East China were 17.20%, 15.99%, 15.62%, 15.33%, 15.07%, 14.87%, and 13.94%, respectively (6). Limited large-scale epidemiological data on AR in Inner Mongolia exist. A 2015 large-scale epidemiological survey by Ma Tingting et al. reported a self-reported AR prevalence of 26.6% among children aged 0–17 years in six grassland regions of Inner Mongolia (44.5% in Xilin Hot, 21.8% in Duolun County, 45.4% in Erenhot, 10.8% in Tongliao City, 27.5% in Zalute Banner, and 15.3% in Kailu County) (7). In 2019, allergen testing of 129 patients with AR from central and western Inner Mongolia (including Hohhot, Baotou, Ulanqab, Ordos and Bayannur) revealed Artemisia muisia as the predominant allergen (8).

Bayannur City situated in the western Hetao Plain of Inner Mongolia Autonomous Region, has a long-term resident population of approximately 1.5 million. Bayannur Meteorological Bureau reports an increase in annual average temperatures and sunshine over the past 60 years, with no significant change in precipitation but a gradual decrease in humidity (9). Artemisia plants are widespread in Bayannur, with artemisia pollen being the major allergen in the Inner Mongolia Autonomous Region of China (7). Recent rapid industrialization and urbanization in Bayannur City have further exacerbated air quality, creating an environment conducive to the spread of AR.

According to the 2023 China Health and Health Statistics Yearbook, Bayannaoer City, with its relatively low economic development, allocates a smaller proportion of its total budget to health expenditures compared to more developed regions. Consequently, per capita health expenditures are below the national average, and attracting public health professionals remains challenging. Additionally, the city's medical and health infrastructure is underdeveloped, resulting in suboptimal treatment options for AR. Currently, the primary methods for treating AR include drug therapy and surgical intervention. For mild symptoms or early-stage cases, hormone + antihistamine therapy is employed alongside nasal cavity irrigation using physiological saline. Severe cases or those with significant nasal septum hypertrophy may require partial resection of the inferior nasal concha. However, specific immunotherapy, including sublingual and subcutaneous allergen immunotherapy widely used globally, is not yet available in Bayannur.

Despite recent large-scale epidemiological studies on AR in Inner Mongolia's major cities, such as Hohhot, Xilin Hot, and Tongliao, detailed prevalence data for Bayannur City remains scarce. Therefore, this study aims to provide a comprehensive analysis of AR prevalence among children aged 6–12 years in Bayannur City, thereby addressing the current gap in epidemiological data for this region.

## 2 Methods

### 2.1 General information

#### 2.1.1 Sample size calculation


$$n\;=\;\frac{t^{2}\mathrm{pq}}{d^{2}}$$

n: sample size; p: Estimated overall prevalence; q = 1-p; d: Tolerance error.

Assuming α is 0.05, t = 1.96, and d = 0.1p, previous studies reported that the prevalence of AR in Inner Mongolia was 17.10%, with an estimated sample size of n = 1,940. To account for the cluster sampling method and to minimize error, the sample size was increased by 50%, resulting in a minimum sample size of 2,929 people.

#### 2.1.2 Random sampling

This cross-sectional study was conducted in Bayannur City, China, which is administratively divided into one district (Linhe District); two counties (Wuyuan County and Dengkou County), and four flags (Urat Front flag, Urat Middle flag, Urat Rear flag, and Hangjin Rear flag). Based on the selection criteria, 89 primary schools, with 75,629 students, were included. Among the 89 primary schools, which were numbered from 1 to 89, six were randomly selected using a lottery method. All children aged 6–12 years from the selected schools who met inclusion criteria were invited to participate. From October 1 to October 15, 2023, electronic QR codes containing informed consent forms and questionnaires were distributed to the parents of the selected children. Parents scanned the QR codes, completed the questionnaires with their children, and submitted them within the specified timeframe. This study was approved by the Ethics Committee of Bayannur City Hospital.

### 2.2 Method

#### 2.2.1 Questionnaire design

The questionnaire consisted of four sections. The first part included the basic information of the children, such as gender, ethnicity, and contact number; The second section was the risk factors, which included information on breastfeeding duration, delivery method, history of food allergies, and other relevant factors. The third section focuses on the diagnosis and classification of AR, while the fourth section addresses the presence of other allergic concomitant diseases.

The reliability of the questionnaire was ensured by referencing international authoritative questionnaires and having it reviewed by experts in allergy science and epidemiology. During the distribution of the questionnaires, rhinologists provided explanations to students and parents. After completion, two study members verified the reliability of the responses. Finally, all responses were imported into an Excel spreadsheet, and after inclusion and exclusion criteria were applied, the eligible samples were coded numerically for statistical analysis.

#### 2.2.2 Partition and diagnosis

Partition: The Land Use Master Plan (2006–2020) provided the basis for area classification (10). Urban areas were considered as concentrated residential zones engaged in industrial, commercial, and service activities. This includes cities, organized towns, development zones, and parks. The agricultural area was characterized by economic activities centered around agriculture, specifically river irrigation farming in the southern Hetao plain, covering approximately 1,147,800 hectares. The pastoral area referred to areas designated for grazing or nomadic activities, including the Wolf Mountain area to the west of the Chashitai Mountain and the northern Urat High Plain, encompassing approximately 10,441,600 hectares. Other areas include small fishing areas, forested areas, and mixed settlements that do not fit into the primary classification.

Diagnosis of AR: Diagnosis was based on the International Study of Asthma and Allergy in Childhood (ISAAC) (11) combined with the Score for Allergic Rhinitis (SFAR) (12). The SFAR scores range from 0 to 16, with a cutoff of 7 points. Scores ≥7 were considered positive for AR, while scores <7 were deemed negative. Participants with positive questionnaire results or a history of positive allergic reactions were classified as AR-positive (Table 1).

**Table 1:** SFAR scores.

Item	Mark
Repeated sneezing, nasal congestion or runny nose in the past year (except when you have a cold)	1 for each symptom 3 total
Rhinoconjunctivitis positive	1
Duration of nasal symptoms	Season 1 Perennial 1
Nasal symptoms trigger	Pollen/dust mites/dust 2 Hair (cat/dog, etc.) 1
Conscious of allergic symptoms	2
Family history of allergies	2
Past positive allergy tests	2
Previous diagnosis of allergic symptoms	1

Classification of AR: Classification followed by the 2019 update of Allergic Rhinitis and its Impact on Asthma (ARIA) guidelines (13). Based on AR severity, it was divided into mild and moderate to severe. Mild was defined as having no symptoms and does not interfere with daily activities and sleep. Moderate to severe was defined as having at least one nasal symptom (runny nose, stuffy nose, itchy nose, or sneezing) that disrupts daily activities and sleep. It can be further divided into continuous and intermittent based on duration. Persistent AR was defined as nasal symptoms that occur more than four days per week for over four consecutive weeks; otherwise, AR is intermittent.

AR concomitant symptoms were defined based on questions. Asthma was determined based on the question “Has your child ever been diagnosed with asthma by a doctor?” The options were Yes or No. Similarly, upper airway syndrome (UACS), secretory otitis media (SOM), sleep apnea-hypopnea syndrome (OSAHS), allergenic dermatitis (AD), and allergic conjunctivitis (AC) were defined.

#### 2.2.3 Inclusion and exclusion criteria

Inclusion criteria: (1) children aged 6–12 years; (2) Residing in Bayannur City for ≥1 year; (3) Parents who have the reading ability and who consent to participate in the survey.

Exclusion criteria: (1) Family members who decline to participate; (2) Inability to accurately assess the child's condition; (3) Incomplete or erroneous questionnaires.

### 2.3 Statistical analysis

SPSS26.0 software was used for data analysis. The Class I error value α was set to 0.05 (both sides). Descriptive analysis for categorical variables was presented as counts and percentages, while quantitative data with a normal distribution were expressed as mean ± standard deviation. For sample size >2,000, the Kolmogorov-Smirnov test was used to evaluate the normality of continuous variables. The Pearson Chi-square test was used to examine the correlation between independent and dependent variables. Univariate logistic regression analysis was performed to identify influencing factors, yielding odds ratios (OR), and 95% confidence intervals (95% CI). Moreover, a correlation was considered statistically significant if *P* < 0.05. Independent variables from univariate analysis were included in multivariate logistic regression analysis, and demographic data including gender, ethnicity, and age were adjusted for Model I. Model II was corrected for breastfeeding duration, delivery mode, history of food or drug allergies, antibiotic use, environmental exposure to smoking, dietary habits, and zoning based on model I.

## 3 Results

### 3.1 Demographic data analysis

#### 3.1.1 Self-reported prevalence of AR

Out of 6 primary schools, 6,225 students were enrolled, and 5,006 questionnaires were collected, yielding an overall response rate of 80.41%. Of these, 4,754 questionnaires were valid, resulting in an effective recovery rate of 94.97%. The sample comprised 2,501 males and 2,253 females, with a male-female ratio of 1.11:1. The prevalence of AR was 39.80% (1,892/4,754), which is higher than reported in other major cities in Inner Mongolia.

#### 3.1.2 AR distribution in the population

The prevalence of AR was 43.06% (1,077/2,501) in males and 2,253 (815/2,253) in females. There were significant differences in AR prevalence between males and females (*X*^2 ^= 23.476, *P* < 0.05). The prevalence of AR was 38.22% (1,524/3,988) in Han nationality and 48.04% (368/766) in minority nationality, with a significant difference observed between these groups (*X*^2 ^= 25.900, *P* < 0.05). The mean age of AR patients was 9.19 ± 1.811, while that of non-AR patients was 9.23 ± 1.860, with no significant difference (*X*^2 ^= 9.127, *P* > 0.05). Gender and nationality significantly affect AR prevalence, but age does not (Table 2).

**Table 2:** Demographic data.

Variable	Total cases	AR cases (%)	*X* ^2^	*P*
Sex			23.476	<0.05
Male	2,501	1,077 (43.06)		
Female	2,253	815 (36.17)		
Nation			25.900	<0.05
The Han nationality	3,988	1,524 (38.22)		
Minority nationality	766	368 (48.04)		
Age				
Average age/years	9.21 ± 1.841	9.23 ± 1.860	9.127	0.164
6	318	201 (63.21)		
7	785	464 (59.11)		
8	652	399 (61.20)		
9	862	497 (57.66)		
10	772	447 (57.90)		
11	678	427 (62.98)		
12	687	427 (62.15)		
Duration of breastfeeding			3.785	0.15
0	492	176 (35.77)		
0–6 month	648	264 (40.74)		
>6 month	3,614	1,452 (40.18)		
Birth			1.270	0.26
Eutocia	2,881	1,128 (39.15)		
Caesarean section	1,873	764 (40.79)		
Food allergy			332.048	<0.05
No	4,072	1,405 (34.50)		
Yes	682	487 (71.41)		
Drug allergy			145.521	<0.05
No	4,156	1,519 (36.55)		
Yes	598	373 (62.38)		
Antibiotic use			71.181	<0.05
No	3,478	1,258 (36.17)		
Frequently	1,276	634 (49.69)		
Exposure to smoking			1.656	0.20
No	2,207	900 (40.80)		
Yes	2,547	992 (38.95)		
Eating habit			8.663	<0.05
Vegetarianism	210	69 (32.86)		
Balanced diet	4,167	1,653 (39.67)		
Meat-Based	377	170 (45.09)		
Subzone			390.001	<0.05
City center	2,312	1,163 (50.30)		
Rural area	750	103 (13.73)		
Pasturing area	445	93 (20.90)		
Other	1,247	533 (42.74)		

AR, allergic rhinitis.

#### 3.1.3 AR general situation correlation analysis

The prevalence of food allergy was 34.50% (1,405/4,072), showing a significant correlation between food allergy and AR prevalence, (*X*^2 ^= 332.048, *P* < 0.05). Drug allergy prevalence was 36.55% (1,519/4,156), also significantly correlated with AR prevalence, (*X*^2 ^= 145.521, *P* < 0.05). The prevalence of AR among those with frequent antibiotic use was 38.95% (634/1,276), with a significant correlation between antibiotic use frequency and AR prevalence (*X*^2 ^= 71.181, *P* < 0.05). Dietary habits revealed that 32.86% (69/32.86) of patients with AR were vegetarian, 39.67% (1,653/4,167) consumed both meat and vegetables, and 45.09% (170/377) had a meat-based diet. A significant correlation was found between dietary habits and AR prevalence (*X*^2 ^= 8.663, *P* < 0.05), with a higher prevalence in meat-based diets. Among these variables, food allergy had the strongest association with AR prevalence (Table 2).

The prevalence of AR in relation to breastfeeding duration was 35.77% (176/492) for <6 months, 40.74% (264/648) for 0–6 months, and 40.18% (1,452/3,614) for >6 months. There was no significant difference in the prevalence of AR with different duration of breastfeeding (*X*^2 ^= 3.785, *P* > 0.05). For different birth modes, the prevalence of AR was 39.15% (1,128/2,881) for vaginal deliveries and 40.79% (764/1,873) for C-sections, with no significant correlation (*X*^2 ^= 1.270, *P* > 0.05). AR prevalence was 62.38% (373/598) in smoking-exposed environments compared to 40.80% (900/2,207) in non-exposed environments, with no significant correlation (*X*^2 ^= 1.656, *P *> 0.05). Breastfeeding time, birth mode, and exposure to smoking were not significant independent risk factors for AR (Table 2).

#### 3.1.4 AR region distribution characteristics

The prevalence of AR was 50.30% (1,163/2,312) in urban areas, 13.73% (103/750) in agricultural areas, 20.90% (93/445) in pastoral areas, and 42.74% (533/1,247) in other areas. AR prevalence was significantly higher in urban areas compared to other regions (*P *< 0.05). Among the four regions, urban areas had the highest impact on AR prevalence (Table 2).

### 3.2 Analysis of risk factors for AR

#### 3.2.1 Single factor logistics regression analysis

Logistic regression analysis was performed on 11 possible related risk factors such as gender, ethnicity, age, breastfeeding time, and the mode of delivery. Male (OR = 1.334, 95% CI = 1.187–1.500), ethnic minority (OR = 1.459, 95% CI = 1.280–1.746), food allergy (OR = 4.747, 95% CI = 3.967–5.666), drug allergy (OR = 2.878, 95% CI = 2.411–3.435), frequent use of antibiotics (OR = 1.743, 95% CI = 1.531–1.948), 50/50 meat and vegetable diet (OR = 1.344, 95% CI = 1.001–1.804) and meat diet (OR = 1.678, 95% CI = 1.1.180–2.378), urban area (OR = 6.358, 95% CI = 5.085–7.949) and pastoral area (OR = 1.660, 95% CI = 1.219–2.260) were potential risk factors for AR (*P *< 0.05). No significant correlation was noted between age, birth mode, breastfeeding time and exposure to a smoking environment and AR (*P* > 0.05) (Table 3).

**Table 3:** Univariate logistic regression analysis of AR in children.

Variable	*OR*	95% CI		*P*
		Lower	Upper	
Sex, male	1.334	1.187	1.500	<0.05
Nation, minority nationality	1.495	1.280	1.746	<0.05
Age				
6	1			0.31
7	1.185	0.902	1.556	0.22
8	1.125	0.849	1.490	0.41
9	1.237	0.945	1.619	0.12
10	1.247	0.949	1.639	0.11
11	1.024	0.773	1.355	0.87
12	1.055	0.798	1.395	0.71
Duration of breastfeeding				
0	1			0.15
0–6 month	1.234	0.969	1.572	0.08
>6 month	1.206	0.991	1.457	0.06
Birth, Caesarean section	1.071	0.951	1.206	0.26
Food allergy, Yes	4.741	3.967	5.666	<0.05
Drug allergy, Yes	2.878	2.411	3.435	<0.05
Antibiotic use, Frequently	1.743	1.531	1.984	<0.05
Exposure to smoking, Yes	0.198	0.825	1.041	0.93
Eating habit				
Vegetarianism	1			<0.05
Balanced diet	1.344	1.001	1.804	<0.05
Meat-based	1.678	1.180	2.387	<0.05
Subzone				
Rural area	1			<0.05
City center	6.358	5.085	7.949	<0.05
Pasturing area	1.660	1.219	2.260	<0.05
Other	4.689	3.702	50,939	<0.05

#### 3.2.2 Multi-factor logistics regression analysis

Multivariate Logistic regression analysis was conducted to evaluate potential risk factors for AR while controlling for confounding variables. Model I was adjusted for gender, ethnicity, and age. Model II, which was based on Model I, was further adjusted for breastfeeding duration, mode of delivery, history of food and drug allergies, antibiotic use, environmental exposure to smoking, dietary habits, and geographic zoning. The statistical results including OR, 95% CI, and *P*-value were analyzed.

The results indicated that the prevalence of AR in males was 1.349 times higher than in females (OR = 1.394, 95% CI = 1.184–1.537, *P *< 0.05). The prevalence was 1.487 times higher in ethnic minorities compared to Han individuals (OR = 1.487, 95% CI = 1.238–1.1.786, *P *< 0.05). Patients with a positive history of food allergies had a prevalence of AR 3.728 times higher than those without (OR = 3.728, 95% CI = 3.049–4.558, *P *< 0.05). A positive history of drug allergies was associated with a 2.093-fold increase in AR (OR = 2.093, 95% CI = 1.693–2.588, *P *< 0.05). The prevalence of AR increased 1.817 times in patients with frequent antibiotic use (OR = 1.817, 95% CI = 1.566–2.108, *P *< 0.05). Compared to rural areas, the prevalence of AR increased 7.292 times in urban areas (OR = 7.292, 95% CI = 5.707–9.317, *P *< 0.05), 1.673 times in pastoral areas (OR = 1.673, 95% CI = 1.199–2.334, *P *< 0.05), and 4.865 times in other areas (OR = 4.865, 95% CI = 3.766–6.286, *P *< 0.05). Therefore, being male, belonging to an ethnic minority, having food or drug allergies, residing in urban or pastoral areas, and exposure to other specific environmental factors were identified as significant independent risk factors (*P* < 0.05), (Table 4).

**Table 4:** Multivariate logistic regression analysis of AR in children.

Influencing factor	Crude OR (95% CI)	Adjust OR (95% CI)	
		Model I	Model Ⅱ
Sex, male	1.334 (1.187–1.500)*	1.350 (1.185–1.537)*	1.349 (1.184–1.537)*
Nation, minority nationality	1.495 (1.280–1.746)*	1.490 (1.240–1.789)*	1.487 (1.238–1.786)*
Food allergy, Yes	4.741 (3.967–5.666)*	3.726 (3.049–4.554)*	3.728 (3.049–4.558)*
Drug allergy, Yes	2.878 (2.411–3.435)*	2.090 (1.691–2.584)*	2.093 (1.693–2.588)*
Antibiotic use, Frequently	1.734 (1.531–1.984)*	1.815 (1.566–2.105)*	1.817 (1.566–2.108)*
Eating habit			
Vegetarianism	1	1	1
Balanced diet	1.334 (1.001–1.804)*	1.151 (0.822–1.611)	1.147 (0.819–1.607)
Meat-based	1.678 (1.180–2.387)*	1.349 (0.904–2.012)	1.346 (0.902–2.010)
Subzone			
Rural area	1	1	1
City center	6.358 (5.085–7.949)*	7.337 (5.757–9.352)*	7.292 (5.707–9.317)*
Pasturing area	1.660 (1.219–2.260)*	1.677 (1.202–2.339)*	1.673 (1.199–2.334)*
Other	4.869 (3.702–5.939)*	4.876 (3.776–6.298)*	4.865 (3.766–6.286)*

Model I: Adjusted for gender, ethnicity, and age.

Model II: On the basis of model I, we adjusted for breastfeeding duration, mode of delivery, history of food allergy, history of drug allergy, antibiotic use, exposure to smoking environment, dietary habits, and zoning.

* *P *< 0.05.

Regarding dietary habits, compared to a vegetarian diet, balanced diets (OR = 1.147, 95% CI = 0.819–1.607) and meat-based diets (OR = 1.346, 95% CI = 0.92–2.010) did not show statistically significant effects on AR prevalence. Hence dietary habits were not considered independent risk factors for AR (Table 4).

### 3.3 Comparison of AR negative, total samples and AR positive three types combined with other allergic diseases

The non-AR group was a sample of children with a negative diagnosis of AR and the presence of other allergic diseases. Specifically: Asthma 4.05% (116/2,862), UACS 14.50% (415/2,862), SOM 2.80% (80/2,862), OSAHS 19.88% (569/2,862), AD 11.76% (337/2,862), AC 6.77% (191/2,862). In the Totality group, there were other instances of allergic disease in the 4,754 valid questionnaires. Asthma 16.70% (797/4,754), UACS 34.33% (1,632/4,754), SOM 10.60% (504/4,754), OSAHS 31.70% (1,507/4,754), AD 26.48% (1,259/4,754), AC 37.93% (1,803/4,754). AR group refers to AR positive at the same time as other allergic diseases. Specifically: Asthma 35.99% (681/1,892), UACS 64.32% (1,217/1,892), SOM 22.41% (424/1,892), OSAHS 49.58% (938/1,892), AD 48.72% (922/1,892), AC 85.20% (1,612/1,892).

**Figure 1:**
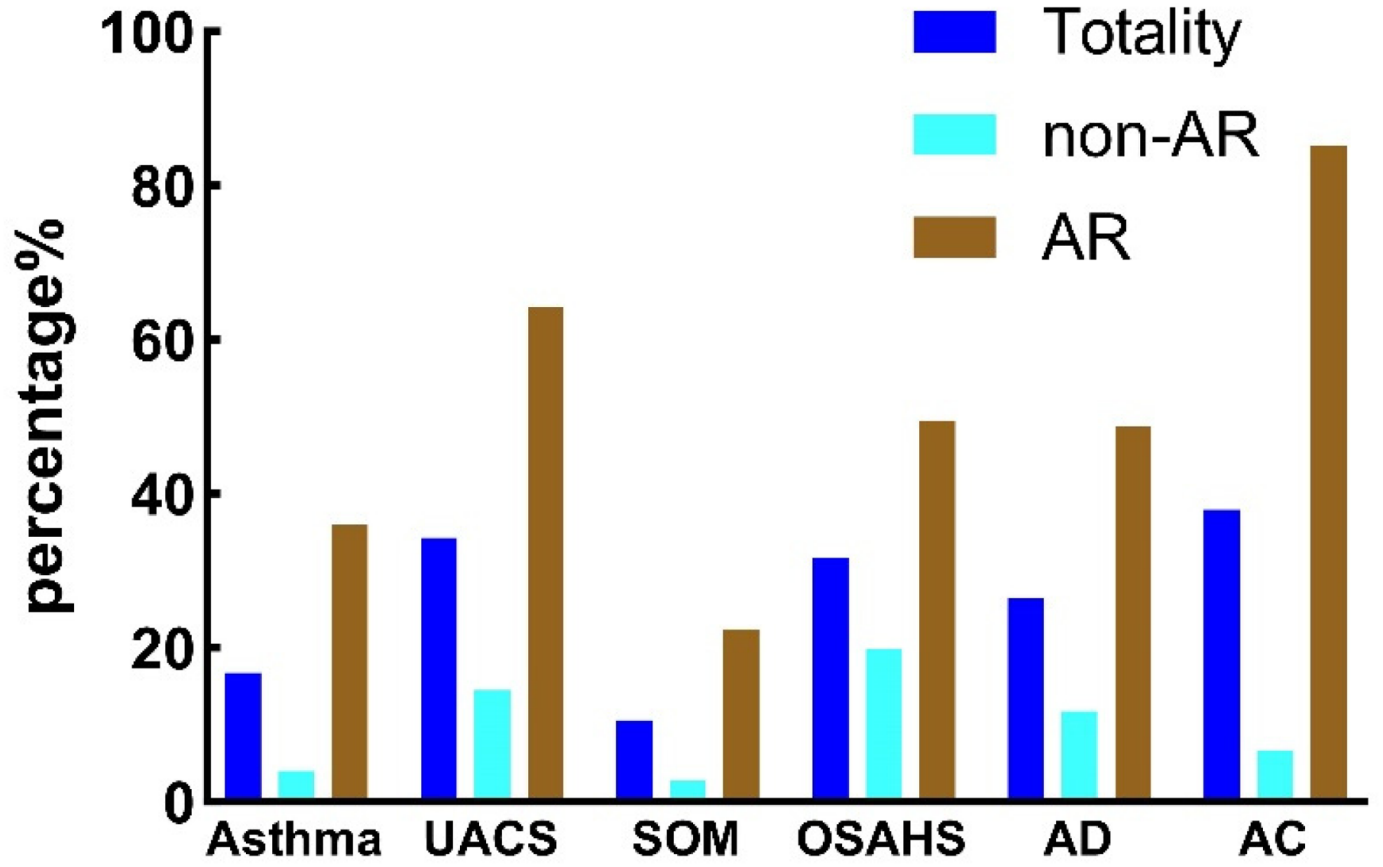
Comparison of samples from different groups with other complications.

The prevalence of concomitant allergic diseases was significantly higher among patients with AR compared to healthy children and the overall sample (*P* < 0.05). Allergic conjunctivitis was the most common comorbidity, affecting 85.20% of patients with AR, while secretory otitis media was less common (Table 5). Compared with the above three groups, the non-AR group exhibited the lowest prevalence of other allergic diseases, with a significant difference from the AR groups. Patients with AR were more likely to suffer from additional allergic diseases (Figure 1).

**Table 5:** Children with other allergic diseases.

Item	Totality	non-AR	AR	*X^2^*	*P*
Asthma	797 (16.7)	116 (4.05)	681 (35.99)	832.742	
UACS	1,632 (34.33)	415 (14.50)	1,217 (64.32)	1,254.177	
SOM	504 (10.60)	80 (2.80)	424 (22.41)	462.384	<0.05
OSAHS	1,507 (31.70)	569 (19.88)	938 (49.58)	463.927	
AD	1,259 (26.48)	337 (11.76)	922 (48.72)	799.023	
AC	1,803 (37.93)	191 (6.67)	1,612 (85.20)	2,862.00	

UACS, upper airway cough syndrome; SOM, secretory otitis media; OSAHS, obstructive sleep apnea hypopnea syndrome; AD, allergic dermatitis; AC, allergic conjunctivitis.

### 3.4 Classification of AR patients

Among the 1,892 patients, 43.29% had intermittent AR, while 56.71% had persistent AR. In terms of severity, 51.22% of patients had mild AR, and 48.78% had moderate to severe AR. Persistent AR was more prevalent, with more than half of the patients experiencing severe symptoms. Thus, AR was more severe in children aged 6–12 years in Bayannur City (Table 6).

**Table 6:** Classification of AR in children.

Item	Number of cases	*X^2^*	*P*
Classification by time		3,362.590	<0.05
Intermittent AR	819 (43.29)		
Persistent AR	1,073 (56.71)		
Classified according to severity		3,294.753	<0.05
Mild AR	969 (51.21)		
Moderate to severe AR	923 (48.78)		

AR, allergic rhinitis.

### 3.5 Prevalence of AR in children in urban, agricultural and pastoral areas

Among 2,312 valid questionnaires, 1,163 were AR-positive, yielding a prevalence rate of 50.30%. Seasonal distribution showed that AR prevalence was 15.48% (180/1,163) from March to May, 54.00% (628/1,163) from June to August, 22.96% (267/1,163) from September to November, and 33.28% (33/1,163) from December to February. A total of 4.73% (55/1,163) of cases did not exhibit clear seasonal patterns. Of the 750 valid questionnaires in rural areas, 103 were AR-positive, giving a prevalence rate of 13.73%. Seasonal distribution in rural areas was 29.13% (30/103) from March to May, 28.16% (29/103) from June to August, 14.56% (15/103) from September to November, and 4.85% (5/103) from December to February, 23.30% (24/103) of cases showing no obvious seasonality. Among 445 valid questionnaires in pastoral areas, 93 were AR-positive, resulting in a prevalence rate of 20.90%. Seasonal distribution in pastoral areas was 13.98% (13/93) from March to May, 46.24% (43/93) from June to August, 21.51% (20/93) from September to November, and 5.38% (5/93) from December to February, with 12.90% (12/93) showing no clear seasonal trend. The prevalence of AR varied significantly across different months in urban areas (Table 7).

**Table 7:** Comparison of AR among children in urban, agricultural and pastoral areas in different seasons.

Variable	City area AR positive (*n* = 1,163)		Rural area AR positive (*n* = 1,163)		Pasturing area AR positive (*n* = 1,163)	
	(*n*)	(%)	(*n*)	(%)	(*n*)	(%)
3–5 month	180	15.48	30	29.13	13	13.98
6–8 month	628	54.00	29	28.16	43	46.24
9–11 month	267	22.96	15	14.56	20	21.51
12–2 month	33	2.84	5	4.85	5	5.38
No concentrated onset period	55	4.73	24	23.30	12	12.90

### 3.6 Comparison of AR in urban, agricultural and pastoral areas in different seasons

**Figure 2:**
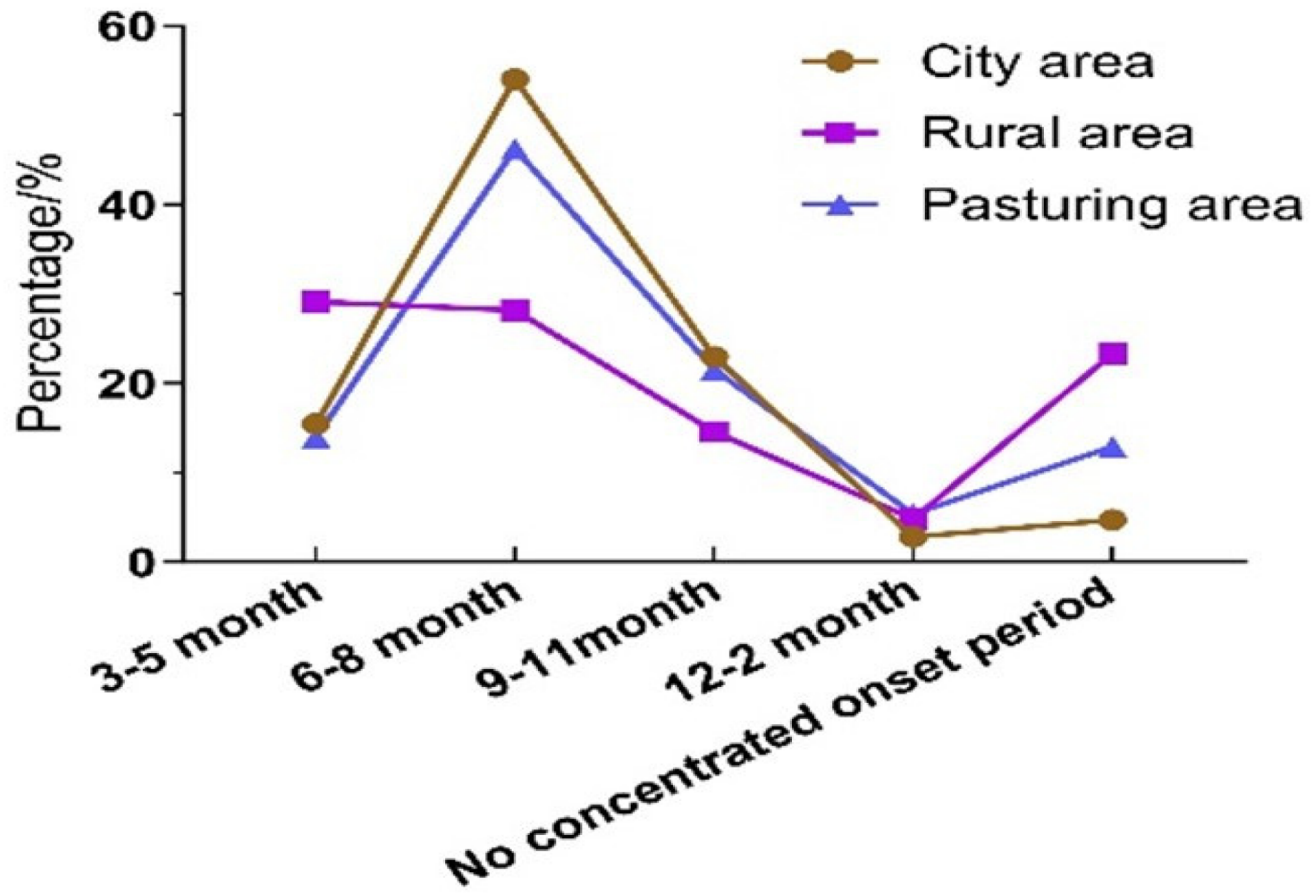
The prevalence of AR in different regions at different times.

The prevalence of AR in urban areas was highest during the summer months from June to August, showing the greatest seasonal variation compared to agricultural and pastoral areas. The epidemic characteristics of AR in pastoral areas and urban areas were similar, but urban areas were more typical of seasonal influences. In contrast, the prevalence rate of AR in rural areas changed slightly in one year. However, the prevalence of AR was highest in rural areas without seasonal effects (Figure 2).

## 4 Discussion

AR is a prevalent chronic disease in children, placing a significant burden on families and posing challenges for healthcare and preventive sectors. The first phase of the ISAAC survey (14), which has been validated and used worldwide, surveyed 700,000 children across 156 centers in 56 countries and reported that developed countries had the highest prevalence of AR among the surveyed countries. The ISAAC Phase 3 study further reported an increase in AR prevalence globally, ranging from 0.8% and 39.7%, affecting both developed and developing countries alike (15). In Europe, the self-reported AR prevalence in the city of Zagreb, Croatia, was 35.70% (16), and 36.2% in Budapest, Hungary (17). In the Middle East, the self-reported prevalence of AR was 39.90% among 851 people in Saudi Arabia (18). The prevalence of AR in children aged 6–18 years in Turkey is 43.20% (19). In Asia, a South Korean study of 12,919 children aged 6–18 revealed a 27.60% increase in AR prevalence over a decade (20), while recent cross-sectional studies in Japan indicate rising rates of nasal conjunctivitis (21). In China, large-scale surveys from 2005 to 2011 across 11 cities revealed significant increases in self-reported AR among adults in eight of these cities (22). Other studies have also reported the regional prevalence of AR in different regions of China. For example, the prevalence of AR among children in Taipei City (23), Xiamen City (24), Zaoyang City (25), and Xilingol League grassland area of Inner Mongolia (26) were 42.80%, 13.70%, 24.31%, and 39.96%, respectively. Given the global rise in AR prevalence, detailed data for Bayannur City are limited. Therefore, this study addressed this gap by conducting a cross-sectional survey of 4,754 children aged 6–12 years in Bayannur City using an electronic questionnaire. The self-reported prevalence rate of AR in children in Bayannur City was 39.80%, which is notably high compared to some developed regions in Europe and Asia, and higher compared to other cities in China.

AR is recognized as the most common allergic disease worldwide, often attributed to a combination of genetic predisposition and environmental factors (27). It involves the abnormal activation of NOD-like receptor thermal protein domain associated protein 3 (NLRP3) and an imbalance in the distribution of CD4-positive cells (Th1), particularly the overexpression of Th2 cells, leading to AR development (28). Our survey, which considered various AR risk factors, revealed through multivariate regression analysis that being male, belonging to an ethnic minority, having a history of food or drug allergies, frequent antibiotic use, and long-term residence are independent risk factors for AR in Bayannur City. A national study in South Korea suggests that sex differences in hormone production and BMI may influence the risk of allergic diseases (29). The higher prevalence of AR among male children in our city might be attributed to such factors. Ethnic minorities also exhibit a higher prevalence of AR. Although there is limited research on racial and ethnic differences in AR, studies by Kim, Yuhree et al. (30) have indicated that individuals of certain non-white races, such as Black and Hispanic populations, experience a higher incidence and persistence of allergic diseases, along with a lower quality of life compared to non-Hispanic whites. Modi et al. (31) also reported significant differences in the effectiveness of subcutaneous allergen immunotherapy (SCIT) across various racial and ethnic groups. A 2010 report highlighted substantial racial and ethnic disparities in health status in the United States, noting that certain diseases are more prevalent in specific populations (32). Therefore, we speculated that these disparities may be due to differences in genetic responses to allergens among ethnic minority individuals compared to Han children, though such studies are lacking. Children with a history of food and drug allergies exhibit a higher prevalence of AR, likely due to heightened sensitivity. The association between frequent antibiotic use and increased AR prevalence is attributed to changes in gastrointestinal flora. Reports suggest that early antibiotic use correlates positively with AR prevalence in children (33).

Additionally, some studies suggest that dietary patterns can influence AR risk. For instance, high vegetable intake and low meat consumption may reduce the risk of AR by decreasing n-6 fatty acid intake (34). Additionally, a study in the United States found that differences in birth patterns between vaginal and cesarean deliveries lead to abnormal microbial colonization or ecological disorders in infancy, potentially affecting allergic disease development (35). Breast milk contains immunoglobulin (Igs), cytokines, and dietary antigens that may regulate immunity. Although direct evidence is lacking, numerous studies have associated breastfeeding duration with allergic disease prevalence in children (36). Research in India has indicated that common indoor air pollutants, such as tobacco smoke, organic compounds from new furniture, and formaldehyde, may increase AR risk in children (37). However, our study found that dietary habits, breastfeeding duration, birth mode, and exposure to smoking are not independent risk factors for AR among children aged 6–12 years in Bayannur City. This suggests that while the independent risk factors for AR are consistent with findings from other regions, the high prevalence observed in Bayannur City may be attributable to unique environmental factors specific to the area.

As previously mentioned, Bayannur City is situated in the grassland region of the northern border of China, between 40°13′–42°28′ north latitude, near the border with Mongolia. Over the past 60 years, air humidity in this region has declined steadily (9). This dry climate may contribute to the high prevalence of AR. Additionally, extensive cultivation of artemisia plants in Bayannur City (38). plays a significant role, as allergen pollen is a major allergen in northern China and contributes substantially to AR prevalence. Epidemiological studies indicate that outdoor air pollution, driven by increased fossil fuel combustion, comprises airway mucous membrane permeability, enhancing allergen penetration and allergic reactions (39). Research conducted in Germany on over 85% of the population revealed that higher degrees of urbanization correlate with increased incidence of respiratory allergic diseases (40). Outdoor air pollutants, including NO_2_, SO2, and particulate matter, are known to elevate AR prevalence in urban areas (41). Moreover, low humidity and water (through hydration) contribute to pollen rupture, which, combined with wind dispersion, increases the concentration of airborne pollen and its allergenic effects (42). Recent industrialization and urbanization in Bayannur City have led to a decline in air quality. According to the Bayannur City Statistics Bureau, industrial production relies predominately on fossil fuels, which constitute 95.30% of energy sources. Therefore, we hypothesized that these environmental factors contribute to the high prevalence of AR among children in Bayannur City. While congenital genetic factors cannot be altered, adjusting children's living conditions and environments could potentially reduce the occurrence of AR.

Studies show that the prevalence of allergic diseases in urban children was significantly higher than that in rural children. Rural living may offer some protection against AR, respiratory allergies, and atopic sensitization (10). Japanese research has also suggested that the height of residence impacts AR prevalence, with bungalows providing a protective effect compared to buildings with 2–5 floors in urban areas (43).This city is situated in the Hetao Plain region of China, characterized by its flourishing agriculture and animal husbandry. Farmers and herdsmen gradually form a breeding state of double baling of grass and livestock and construction of net fences, and their living environment is different from that of urban areas (44). According to the seventh national census, the city is home to over 110,000 ethnic minorities, including 85,000 Mongolians. This concentration of livestock production and pastoral area contributes to a unique environment. The medical services available to residents in Bayannur's farming and pastoral areas are relatively underdeveloped, with limited health education and resources. This lack of access to medical care and education is reflective of a broader global issue, where disparities exist between urban and rural health resources. Based on the characteristics of the natural environment, the distribution of population characteristics, and the low resources of medical and health care in this region, we further studied the prevalence of AR among children aged 6–12 years in urban, agricultural, and pastoral areas. Our study found that the prevalence of AR among children aged 6–12 years in urban areas was higher compared to those in pastoral and agricultural areas. This observation aligns with the understanding that rapid urbanization contributes to increased AR prevalence. However, our findings also revealed a higher prevalence of AR in pastoral areas compared to rural areas, thereby contributing to the global data on AR prevalence in rural and pastoral settings.

Leynaert et al. have shown a positive correlation between the severity and duration of AR and the prevalence of asthma (45, 46). Additionally, Japanese researchers have identified olfactory dysfunction in children with moderate to severe AR (47). Chronic AR patients are also at a higher risk for depression, bipolar disorder, and increased suicidal tendencies (4). Antonella Gambadauro et al. found that AR is linked not only to attention deficit hyperactivity disorder (ADHD) but also that patients with severe AR combined with ADHD exhibit better compliance with AR treatment than those with mild AR. This compliance is inversely correlated with inattention symptoms in children with ADHD (48). This compliance is inversely correlated with inattention symptoms in children with ADHD. Thus, addressing emotional and behavioral aspects is crucial in managing AR effectively, particularly for the 48.78% of children in our study with server AR.

The abnormal activation of NLRP3 contributes to AR and other type 2 inflammatory conditions, including asthma, atopic dermatitis, and various respiratory, gastrointestinal, and skin diseases (49, 50). Consequently, AR patients are often accompanied by other allergy symptoms. Reports indicate that 19%–38% of patients with AR may have asthma, and among patients with both diseases, more than 75% develop the secondary condition within two years if not concurrently present (51). Furthermore, AR is recognized as a significant risk factor for otitis media effusion, alongside bacterial infection and eustachian tube obstruction (52). In our study, only 14.709% of children with AR in Bayannur City did not have any concurrent allergic conditions, aligning with global trends. Therefore, parents and medical personnel need to monitor the presence of other allergic comorbidities while actively preventing and treating AR.

To address the high incidence of AR among children in Bayannur City, several preventive and medical recommendations are proposed. Bayannur City should consider vegetation cover instead of wormwood to reduce the main allergens. Concurrently, efforts to transition from fossil fuels to cleaner energy sources could help reduce air pollution. Moreover, public health campaigns should emphasize preventive measures such as wearing masks and goggles to minimize allergen exposure, promoting regular physical exercise and a healthy diet, and reducing the early and frequent use of antibiotics to improve immunity. Children and parents should also be encouraged to seek medical attention when AR or accompanying symptoms are observed initially to avoid worsening symptoms. For AR treatment, current practices in Bayannur City primarily involve medication and surgery. However, the introduction of specific immunotherapy and desensitization therapy should be pursued to help patients build tolerance to allergens and alleviate symptoms. Future advancements might include gene modification therapies to address hypersensitivity comprehensively. These strategies could serve as a model for other areas with similar medical and environmental conditions.

It should be noted that there are limitations to this study. This study is a large-scale epidemiological survey, and the sample size was expanded to minimize sampling error and potential selection bias. The conclusions drawn are based on self-reported data, which, while extensive, could be enhanced by incorporating physical examinations by specialists and laboratory examinations for allergen detection. These additional measures would improve the accuracy of the prevalence data.

This is an area for future research to obtain more precise data on AR prevalence among children in this region.

To mitigate the occurrence of deviation, we also implemented relevant measures. First, the sample size was increased, and random sampling was employed. All team members underwent professional training before distributing questionnaires. They emphasized the anonymity, lack of innovation, and importance of the questionnaire when they visited schools in person. The contents of the questionnaire were explained in detail, and support from the municipal Education Bureau was secured to bolster the trust and attention of children and parents. Second, the questionnaire included a verification question: “In the past year, have you or your child been diagnosed with allergic rhinitis by a healthcare provider?”. This question helped validate some of the responses. Finally, after completing the report, the team members contacted 10% of the parents by telephone to verify the accuracy of the data. The questionnaire results were verified against medical records from local hospitals to minimize errors related to questionnaire completion.

## 5 Conclusions

Not only in Inner Mongolia or China, children aged 6–12 in Bayannur City have a high prevalence of AR in the world. And nearly half of the symptoms were severe enough to interfere with daily life. Prevention departments and medical and health institutions should strengthen the prevention and treatment of AR in the region. In addition to genetic factors, we preliminarily speculate that the most influential factor of AR in this region is the natural environment. Therefore, we call on the government departments to target the reduction of wormwood in the future development, while reducing the use of fossil fuels and improving air quality. This study is the first large-scale epidemiological investigation on children's AR in Bayannur City, and we hope that the data from this study can provide a data basis for the epidemiology of children's AR in China. At the same time, we also hope that this study will increase attention to the current prevalence of childhood AR in grassland regions around the world.

## Data Availability

The datasets presented in this study can be found in online repositories. The names of the repository/repositories and accession number(s) can be found in the article/Supplementary Material.
