# Supplementary material for: Epidemiological investigation of allergic rhinitis in children aged 6–12 years in Bayannur City, China
Source: Front Pediatr. 2024 Sep 24;12:1422323. doi: 10.3389/fped.2024.1422323 (PMC11458438; doi:10.3389/fped.2024.1422323)
Supplement: Supplementary file 1 [file Datasheet1.pdf]

Questionnaire on allergic rhinitis in children aged 6-12 years

Demographic data

1. Gender: ○ Male ○ female

2. Age: ______ years old. ○6 ○7 ○8 ○9 ○10 ○11 ○12

3. Ethnic Group: ○ Han ○ Minority

4. Residence area (⩾1 year) : ______ (accurate to community)

5. Duration of breastfeeding ____. ○0 ○0-6 months ○ >6

6. Birth____. ○Eutocia ○Caesarean section

7. History of food allergy____. ○No ○Yes

8. Drug allergy history____. ○No ○Yes

9. Frequent use of antibiotics (⩾3 times per year for ⩾3 days in the past two years) ____. ○No ○Yes

10. The type of diet you usually eat ____. ○Vegetarianism ○Eat a balanced diet ○Meat-Based

Clinical symptoms of allergic rhinitis

1. Have you or your child sneezed repeatedly (except when you have a cold) ____. ○No ○Yes

2. Have you or your child had recurring nasal congestion (except when they have a cold) ____. ○No ○Yes

3. Have you or your child had a recurring runny nose (except when a cold) in the past year ____. ○No ○Yes

4. Whether the nasal symptoms are accompanied by eye symptoms such as itching and tearing ____. ○No ○Yes

5) Months of nasal symptoms (can be selected) ____.

○3-5 ○6-8 ○9-11 ○12-2 ○None in the year

6. There are triggers for nasal symptoms ____.

○Dust, pollen, etc ○hair（cat and dog, etc）

7. 1. In the past year, have you or your child experienced allergies____.

○No ○Yes

8.In the past year, have you or your child been diagnosed with allergic rhinitis by a healthcare provider____. ○No ○Yes

9. Had a positive allergy test (skin prick test, serological specific IgE test and other types of tests) ____. ○No ○Yes

10. Whether someone in your family (Father, mother, brother/sister, grandparent or grandparent) has an allergic disease____. ○No ○Yes

11. Onset time of nasal symptoms ____. ○No ○< 4 days/week, or symptom duration < 4 weeks ○> 4 days/week, or symptom duration > 4 weeks

12. Severity of nasal symptoms: ____. ○No ○The symptoms are mild and have no significant impact on the quality of life (daily life, learning, sleep, exercise, etc.) ○The symptoms are more severe or very severe, and have a significant impact on the quality of life (daily life, learning, sleep, exercise, etc.)

Concomitant symptom

1.Has your child ever been diagnosed with asthma by a doctor____. ○No ○Yes

2. Has your child ever been diagnosed with upper airway cough syndrome ____.

○No ○Yes

3.Has your child ever been diagnosed with secretory otitis media____. ○No ○Yes

4. Has your child ever been diagnosed with sleep apnea hypopnea syndrome by a doctor____. ○No ○Yes

5. Has your child ever been diagnosed with atopic dermatitis ____. ○No ○Yes

6. Has your child ever been diagnosed with allergic conjunctivitis ____.

〇No　〇Yes
